# Vitamin D deficiency in Malawian adults with pulmonary tuberculosis: risk factors and treatment outcomes

**DOI:** 10.5588/ijtld.15.0071

**Published:** 2015-08

**Authors:** D. J. Sloan, H. C. Mwandumba, M. Kamdolozi, D. Shani, B. Chisale, J. Dutton, S. H. Khoo, T. J. Allain, G. R. Davies

**Affiliations:** * Malawi Liverpool Wellcome Trust Clinical Research Programme, College of Medicine, University of Malawi, Blantyre, Malawi; † Liverpool Heart and Chest Hospital, Liverpool; ‡ Liverpool School of Tropical Medicine, Liverpool, United Kingdom; § Department of Microbiology, College of Medicine, University of Malawi, Blantyre; ¶ Department of Medicine, College of Medicine, University of Malawi, Blantyre, Malawi; # University of East Anglia, Norwich; ** Department of Pharmacology, University of Liverpool, Liverpool; †† Institute of Infection and Global Health, University of Liverpool, United Kingdom

**Keywords:** treatment failure, relapse, seasonality, HIV, antiretroviral therapy

## Abstract

**SETTING:** Vitamin D deficiency is common in African adults with tuberculosis (TB), and may be exacerbated by the metabolic effects of anti-tuberculosis drugs and antiretroviral therapy (ART). It is unclear whether vitamin D deficiency influences response to anti-tuberculosis treatment.

**OBJECTIVES:** To describe risk factors for baseline vitamin D deficiency in Malawian adults with pulmonary TB, assess the relationship between serum 25-hydroxy vitamin D (25[OH]D) concentration and treatment response, and evaluate whether the administration of anti-tuberculosis drugs and ART is deleterious to vitamin D status during treatment.

**DESIGN:** A prospective longitudinal cohort study.

**RESULTS:** The median baseline 25(OH)D concentration of the 169 patients (58% human immunodeficiency virus [HIV] infected) recruited was 57 nmol/l; 47 (28%) had vitamin D deficiency (<50 nmol/l). Baseline 25(OH)D concentrations were lower during the cold season (*P* < 0.001), with food insecurity (*P* = 0.034) or in patients who consumed alcohol (*P* = 0.019). No relationship between vitamin D status and anti-tuberculosis treatment response was found. 25(OH)D concentrations increased during anti-tuberculosis treatment, irrespective of HIV status or use of ART.

**CONCLUSIONS:** Vitamin D deficiency is common among TB patients in Malawi, but this does not influence treatment response. Adverse metabolic effects of drug treatment may be compensated by the positive impact of clinical recovery preventing exacerbation of vitamin D deficiency during anti-tuberculosis treatment.

## RESUME

**CONTEXTE :** Le déficit en vitamine D est fréquent chez les adultes africains atteints de tuberculose (TB), et il peutêtre exacerbé par les effets métaboliques des médicaments antituberculeux et par la thérapie antirétrovirale (ART). On ne sait pas vraiment si le déficit en vitamine D influence la réponse au traitement de la TB.

**OBJECTIF: D**écrire les facteurs de risque du déficit en vitamine D initial chez des adultes du Malawi atteints de TB pulmonaire,évaluer la relation entre la concentration sérique en 25 hydroxyvitamine D (25[OH]D) et la réponse au traitement etévaluer si l'administration de médicaments antituberculeux et l'ART ont un effet négatif sur le statut de la vitamine D au cours du traitement.

**SCHÉMA:** Etude de cohorte prospective longitudinale.

**RÉSULTATS:** Ontété recrutés 169 patients (58% infectés par le virus de l'immunodéficience humaine [VIH]). La concentration médiane initiale du 25(OH)D aété de 57nmol/l ; 47 (28%) patients ont eu un résultat <50 nmol/l, c'est-à-dire, un déficit en vitamine D. Les concentrations initiales de 25(OH)D ontété plus faibles pendant la saison froide (*P* < 0,001), avec l'insécurité alimentaire (*P* = 0,034) ou chez des patients qui consommaient de l'alcool (*P* = 0,019). Il n'y a pas eu de relation entre le statut en vitamine D et la réponse au traitement de la TB. Les concentrations de 25(OH)D se sont accrues durant la thérapie antituberculeuse, quel que soit le statut VIH ou le recoursà l'ART.

**CONCLUSIONS:** Le déficit en vitamine D est fréquent chez les patients tuberculeux au Malawi, mais il n'influence pas la réponse au traitement. Les effets secondaires métaboliques des traitements médicamenteux pourraientêtre compensés par l'impact positif de la guérison clinique qui prévient une aggravation du déficit en vitamine D pendant le traitement de la TB.

## RESUMEN

**MARCO DE REFERENCIA:** La deficiencia de vitamina D es frecuente en los adultos que padecen tuberculosis (TB) enÁfrica y su exacerbación puede ser la consecuencia de efectos metabólicos provocados por los medicamentos antituberculosos y el tratamiento antirretrovírico (ART). No es claro si esta deficiencia vitamínica influye sobre la respuesta al tratamiento de la TB.

**OBJETIVOS:** Describir los factores de riesgo de los pacientes adultos con TB pulmonar en Malawi de sufrir una deficiencia de vitamina D al comienzo del estudio, evaluar la correlación entre la concentración sérica de 25 hidroxi-vitamina D (25[OH]D) y la respuesta al tratamiento y analizar si la administración de medicamentos antituberculosos o ART altera el estado de la vitamina D durante el tratamiento.

**MÉTODO:** Fue este un estudio de cohortes longitudinal prospectivo.

**RESULTADOS:** Participaron en el estudio 169 pacientes (58% con coinfección por el virus de la inmunodeficiencia humana [VIH]). La mediana de la concentración sérica de 25(OH)D al comienzo del estudio fue 57 nmol/l. Se encontró deficiencia de vitamina D en 47 pacientes (28%; <50 nmol/l). Las concentraciones séricas de base fueron más bajas durante la temporada fría (*P* < 0,001), cuando existía inseguridad alimentaria (*P* = 0,034) o en los pacientes que consumían alcohol (*P* = 0,019). No se observó una correlación entre la concentración de la vitamina D y la respuesta al tratamiento antituberculoso. Las concentraciones séricas de 25(OH)D aumentaron durante el tratamiento antituberculoso, independientemente de la situación frente al VIH o la administración del ART.

**CONCLUSIÓN:** La deficiencia de vitamina D es frecuente en los pacientes con diagnóstico de TB en Malawi, pero esta carencia no afecta su respuesta al tratamiento. Los efectos metabólicos de los medicamentos se compensan con el efecto positivo de la recuperación clínica, lo cual evita una exacerbación de la deficiencia vitamínica durante el tratamiento de la TB.

TUBERCULOSIS (TB) remains a major public health problem in Malawi. In 2013, 156 cases were notified per 100 000 population, and 56% of TB patients were human immunodeficiency virus (HIV) co-infected.^1^ Despite the consistent implementation of World Health Organization (WHO) approved treatment, successful outcomes from the Malawian National Tuberculosis Control Programme (NTP) have remained at ~80% for the last decade.^1^,^2^ Risk factors contributing to poor outcomes for individual patients, and strategies to ameliorate these risk factors, have not yet been identified.

Previous work has shown that vitamin D deficiency is more common in Malawian adults with TB than in the general hospital population.^2^,^3^ The most active vitamin D metabolite is 1,25 hydroxycholecalciferol (1,25[OH]D), an immunologically active hormone that stimulates antimycobacterial activity in vitro.^4–9^ Although clinical trials have not shown improved clinical outcomes when standard anti-tuberculosis treatment is augmented with oral vitamin D,^10–12^ target serum levels of vitamin D for a clinically significant effect are unknown, and some researchers advocate alternative dosing strategies or describe benefits for particular patient groups.^10^ A single study from Tanzania reported that low baseline serum vitamin D concentrations were associated with poor clinical outcomes.^13^

Furthermore, some components of anti-tuberculosis treatment and antiretroviral therapy (ART) may pharmacologically lower serum concentrations of useful vitamin D metabolites. Vitamin D is synthesised in the skin after exposure to sunlight or consumed in the diet, then converted by sequential hydroxylation into 25(OH)D and 1,25(OH)D. Isoniazid (INH) inhibits both hydroxylation steps,^14^ while rifampicin (RMP) induces alternative enzyme activity to degrade 25(OH)D into a waste product.^15^ Combined RMP and INH treatment may reduce serum concentrations of useful vitamin D metabolites by 23–34%.^15^ Some ART drugs, such as efavirenz (EFV), have also been associated with vitamin D deficiency.^16^ It is therefore possible that low baseline vitamin D levels are further compromised by drug therapy in HIV-TB co-infected populations.

The present study aimed to describe risk factors for baseline vitamin D deficiency in a cohort of Malawian adults with pulmonary TB, assess the relationship between 25(OH)D concentrations and treatment response, and evaluate whether the administration of RMP, INH and ART is deleterious to vitamin D status during treatment.

## METHODS

### Patient recruitment and follow-up

This work was nested within a longitudinal cohort study of response to anti-tuberculosis treatment among Malawian adults at Queen Elizabeth Central Hospital in Blantyre, Malawi, from 2010 to 2012. Consenting adults with sputum smear-positive pulmonary TB graded ‘++’ or ‘+++’ for acid-fast bacilli (AFB) on Ziehl-Neelsen (ZN) stained slides were eligible.^17^ Exclusion criteria included haemoglobin <6 g/dl, creatinine >177 μmol/l, total bilirubin >51 μmol/l, alanine transaminase >200 international units (IU)/l, clinical status suggestive of imminent mortality (WHO performance score 4^18^), pregnancy, anti-tuberculosis treatment in the last 5 years, corticosteroid therapy or baseline resistance to RMP and INH. All patients underwent chest radiograph (CXR) and point-of-care HIV serology. ART was available according to national protocols.^19^,^20^ Anti-tuberculosis treatment was prescribed according to NTP guidelines:^21^ RMP, INH, pyrazinamide and ethambutol were administered for 8 weeks, followed by RMP and INH for 16 weeks. Follow-up continued for 1 year after end of treatment to incorporate relapse rates into final outcomes.

Patients with negative TB sputum cultures from the end of treatment onwards or who stopped coughing and remained well until study discharge were defined as ‘stable cures’. Those who were culture-positive at the end of treatment were ‘failures’. Those who were culture-negative at the end of treatment, but who subsequently re-developed positive cultures were ‘relapses’. Final treatment outcome was defined as the composite ‘unfavourable’ clinical endpoint of failure or relapse.

### Vitamin D measurement

Serum samples were collected from each patient at baseline, week 8 and the end of treatment, and stored at −70°C until analysis in a single batch. Briefly, 25(OH)D_2_ and D_3_ were extracted using zinc sulphate and acetonitrile as precipitants. Samples were centrifuged to obtain a supernatant. Bio-analysis was performed using reverse phase liquid chromatography coupled to a tandem mass spectrometer in electro spray ionisation positive mode. Quantification of 25(OH)D_2_ and 25(OH)D_3_ metabolites was based on multiple reaction monitoring of the specific mass transition for each target analyte. Total 25(OH)D was the sum of (OH)D_2_ and (OH)D_3_ at each timepoint. Holick's definitions of vitamin D status were used:^22^ hypovitaminosis D if 25(OH)D ⩽75 nmol/l, vitamin D deficiency if ⩽50 nmol/l, and severe vitamin D deficiency if ⩽25 nmol/l.

### Sputum bacteriology

Baseline sputum samples were assessed using ZN and auramine phenol smear microscopy^17^ to confirm smear positivity, and were set up for culture using solid media and liquid broth. For solid media, 1 ml of undecontaminated sputum was homogenised with an equal volume of dithiothreitol (Oxoid, Basingstoke, UK), and five serial ten-fold dilutions were prepared in phosphate buffered saline; 50 μl of neat sputum and each dilution were plated onto duplicate plates of Middlebrook 7H11 (BD, Sparks, MD, USA) oleic-acid albumin agar media made selective by the addition of polymyxin B (200 U/ml), ticarcillin (100 mg/l), trimethoprim (10 mg/l) and amphotericin B (10–30 mg/l). After 3 weeks of incubation, visible Mycobacterium tuberculosis colonies denoted positive cultures and dilutions yielding 10–100 colonies were selected for counting. The baseline bacillary load was the average log_10_ colony forming unit/ml of sputum from duplicate plates of each sample.

One ml of each sputum sample was decontaminated using *N*-acetyl-L-cysteine/sodium hydroxide 3% and inoculated into MGIT^™^ (Mycobacterial Growth Indicator Tube, BD, Sparks, MD, USA). These were placed in an incubator at 37°C until they signalled positive. The GenoType^®^ MTBDR*plus* 2.0 line-probe assay (Hain Life Sciences, Nehren, Germany) confirmed that all M. tuberculosis isolates were RMP-and INH-susceptible. Samples that did not signal positive at 7 weeks were regarded as negative.

Sputum samples collected at 8 weeks, at end of treatment and during post-treatment follow-up were used to assess 2-month smear/culture conversion and allocate final outcomes. All specimens were inoculated onto solid media and into broth, and were reported as culture-positive if M. tuberculosis grew by either method. Those with no growth in any media were reported as negative.

### Data analysis and statistical methods

Data were described using non-parametric summary statistics. Multivariate linear regression was used to assess factors contributing to variability in baseline serum 25(OH)D. A linear trapezoid rule was used to calculate an area under the concentration time curve (AUC_0–6 months_) for each patient, representing total 25(OH)D exposure during anti-tuberculosis treatment. Logistic regression was used to study relationships between baseline 25(OH)D and treatment response (2-month smear or culture status and final outcome). Relationships between changes from baseline concentration during treatment or AUC_0–6 months_ and final outcome were also evaluated. Results of linear and logistic regression analyses were expressed as regression coefficients or odds ratios (ORs) with 95% confidence intervals (CIs). The Kruskal-Wallis test was used to assess variability in baseline 25(OH)D at different recruitment months, and AUC_0–6 month_ variability among patients who initiated ART at different stages of anti-tuberculosis treatment. Changes in 25(OH)D at different time-points were analysed using paired Wilcoxon tests. Significance was reported at *P* < 0.05.

### Ethics

Ethics approval for this study was granted by the Liverpool School of Tropical Medicine, Liverpool, UK, and the College of Medicine Research Ethics Committee, University of Malawi, Blantyre, Malawi. Written informed consent was provided by all participants.

## RESULTS

### Patients and outcomes

Baseline characteristics of the 169 patients recruited are outlined in the Table. The median age was 31 years; 116 (69%) were male. The median body mass index (BMI) was 18.4 kg/m^2^, and 33 (20%) patients reported food insecurity (defined as regularly missing more than one meal per day in the last month). Of the 169 patients, 52 (36%) had cavities on CXR; 98 (58%) were HIV-infected, with a median CD4 count of 163 cells/μl.

**Table:**
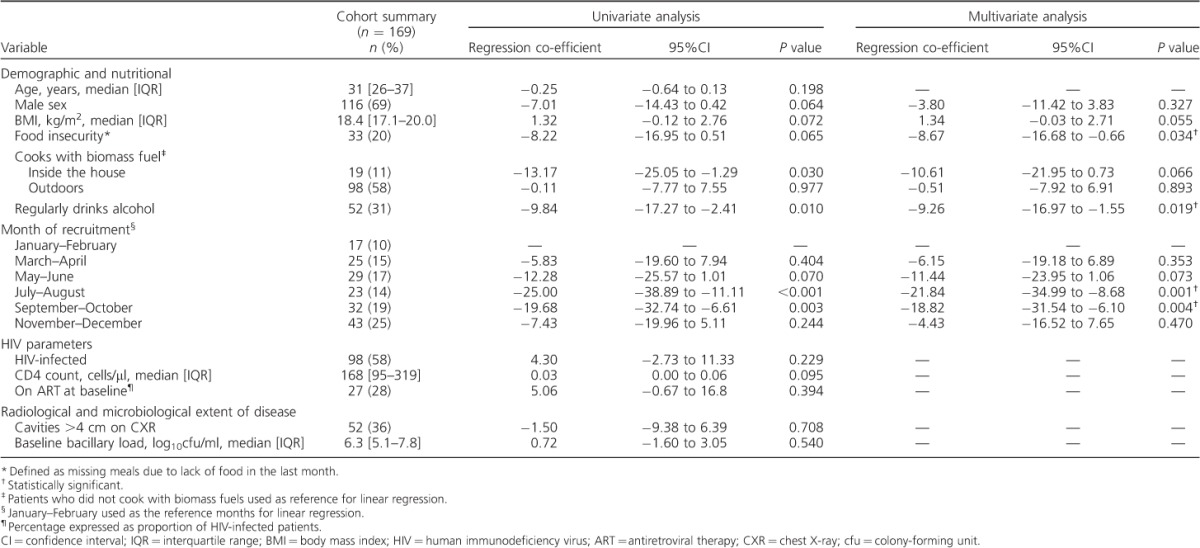
Variables associated with baseline serum vitamin D concentration in adult Malawian patients with pulmonary tuberculosis

**Figure 1.**
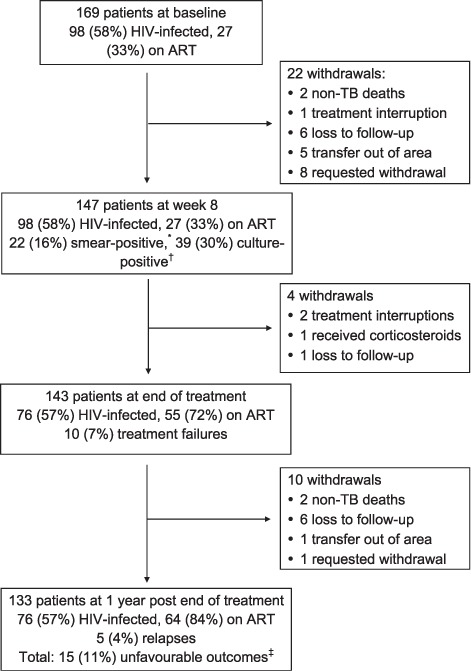
Patient retention, progress and outcomes.* 8-week sputum smear results available for 141 patients.^†^ 8-week sputum culture results available for 131 patients.^‡^ Unfavourable outcomes = failures at end of treatment (*n* = 10) + post-treatment relapses (*n* = 5). HIV = human immunodeficiency virus; ART = antiretroviral therapy; TB = tuberculosis.

Figure 1 shows patient retention and progress through treatment of the study participants. At 8 weeks, 147 individuals remained in the study. For those with sputum smear and culture results at this time, 22/141 (16%) patients remained smear-positive and 39/131 (30%) remained culture-positive. Overall, 133 patients remained in the study until allocation of a final outcome: 118 (89%) achieved stable cure, while 15 (11%) had unfavourable outcomes.

At baseline, 27/98 (33%) HIV-infected patients were undergoing ART. By study discharge, 64/76 (84%) HIV-infected patients were undergoing ART. The ART regimen among study completers was as follows: 57 (89%) received stavudine (d4T), lamivudine (3TC) and nevirapine (NVP), 1 (2%) received zidovudine, lamivudine and nevirapine, 3 (5%) received tenofovir (TDF), 3TC and EFV and 3 (5%) were started on d4T, 3TC and NVP but switched to TDF, 3TC and EFV during anti-tuberculosis treatment.

### Vitamin D status at baseline

**Figure 2.**
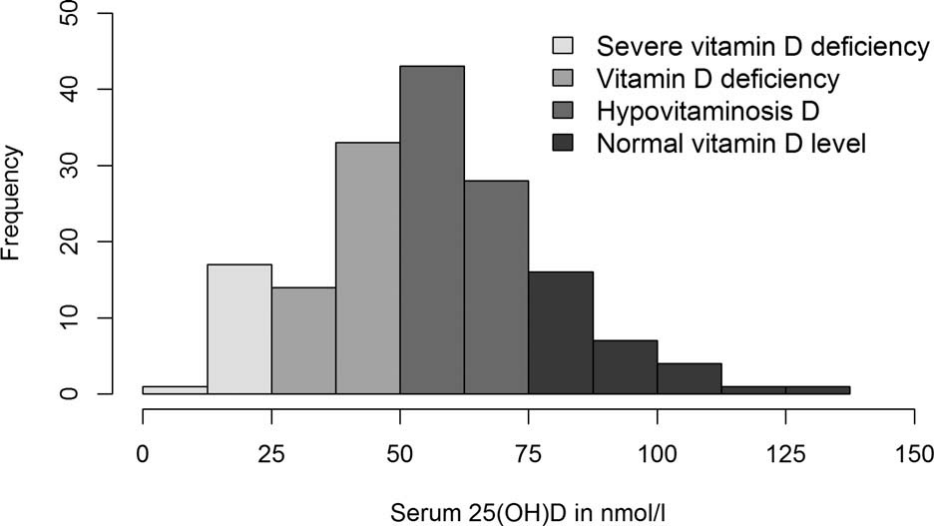
Histogram of baseline serum 25(OH)D concentrations. Of 166 patients with serum 25(OH)D concentrations measured at baseline, 29 (18%) had results within the normal range and 72 (43%) had hypovitaminosis D; 65 (39%) had vitamin D deficiency, 18 (11%) of whom had severe deficiency.

Baseline serum 25(OH)D concentrations were available for 166 patients, with a median value of 57 nmol/l: 29 (18%) patients had 25(OH)D within the normal range and 72 (43%) had hypovitaminosis D; 65 (39%) had vitamin D deficiency, 18 (11%) of whom had severe deficiency. The distribution of 25(OH)D measurements throughout the study population is shown in Figure 2.

**Figure 3.**
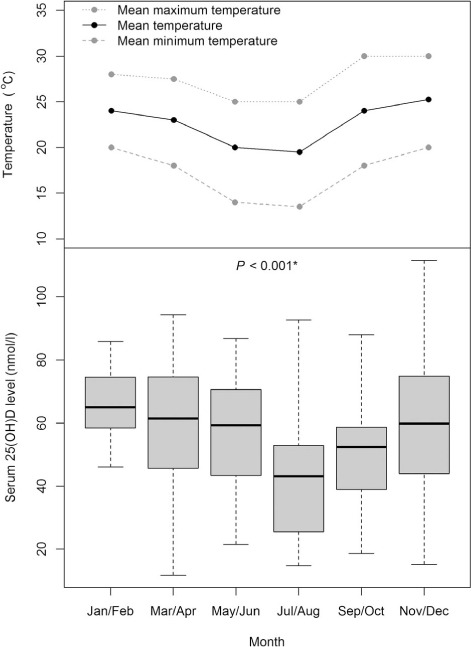
Effect of seasonality on baseline serum 25(OH)D concentration. Serum 25(OH)D concentrations from the 166 patients with baseline data were lower in study participants recruited during or just after the cold season. The *P* value showing seasonal differences in serum 25(OH)D concentrations was derived from the Kruskal-Wallis test.

The Table and Figure 3 show that, on multivariate analysis, the strongest factor associated with baseline vitamin D status was the month of recruitment; participants recruited in July/August or September/October had lower serum 25(OH)D than those recruited in January/February (*P* = 0.001 and *P* = 0.004, respectively). Lower 25(OD) also occurred in patients with food insecurity (*P* = 0.034) and those who regularly consumed alcohol (*P* = 0.019). There were trends towards lower concentrations in patients with lower BMI (*P* = 0.055) or who cooked indoors with biomass fuel (*P* = 0.066), but no relationships between vitamin D status and HIV infection parameters, CXR cavitation or baseline bacillary load.

**Figure 4.**
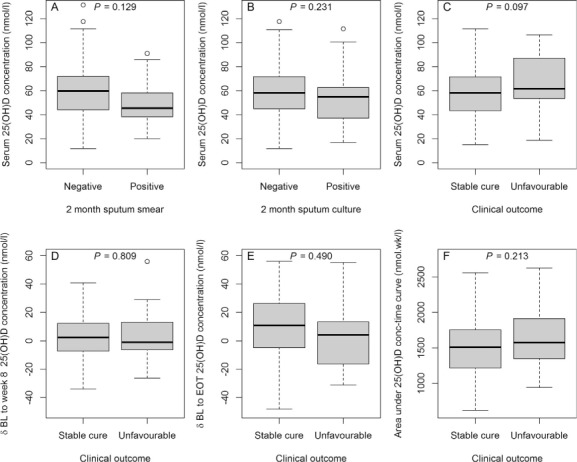
Variability in serum 25(OH)D concentrations by response to anti-tuberculosis treatment: **A–C)** difference in baseline serum 25(OH)D concentration by 2-month sputum smear status (*n* = 141), 2-month sputum culture status (*n* = 131) and final clinical outcome (*n* = 133), respectively; **D)** changes in 25(OH)D concentration from baseline to 8 weeks; **E)** changes in 25(OH)D concentration from baseline to end of treatment by clinical outcome (*n=*133); **F)** 25(OH)D exposure across the duration of treatment, represented by the area under the concentration time curve by clinical outcome (*n* = 133). All *P* values are derived from logistic regression analysis.

Figure 4 illustrates that, on univariate analysis, there was no significant relationship between baseline 25(OH)D and the likelihood of a positive 2-month sputum smear (OR 0.98, 95%CI 0.96–1.00, *P* = 0.129) or a positive 2-month sputum culture (OR 0.99, 95%CI 0.97–1.01, *P* = 0.231). Surprisingly, there was a slight trend towards unfavourable final outcomes at higher baseline 25(OH)D (OR 1.02, 95%CI 1.00–1.05, *P* = 0.097). As the effect size was very small and statistical significance was not reached, it is unlikely that this was clinically relevant. When vitamin D status was analysed as a categorical variable with cut-offs at 25, 50 or 75 nmol/l, there were no associations with any markers of treatment response (data not shown). Multivariate models were also constructed to establish whether incorporation of other variables from the Table influenced response to treatment. Although advancing age (OR 1.12, 95%CI 1.03–1.22, *P* = 0.008) was independently associated with positive 2-month sputum smears, all multivariate models were consistent with the conclusion from Figure 4 that there were no significant relationships between 25(OH)D concentrations and treatment response.

### Vitamin D status during anti-tuberculosis treatment

**Figure 5.**
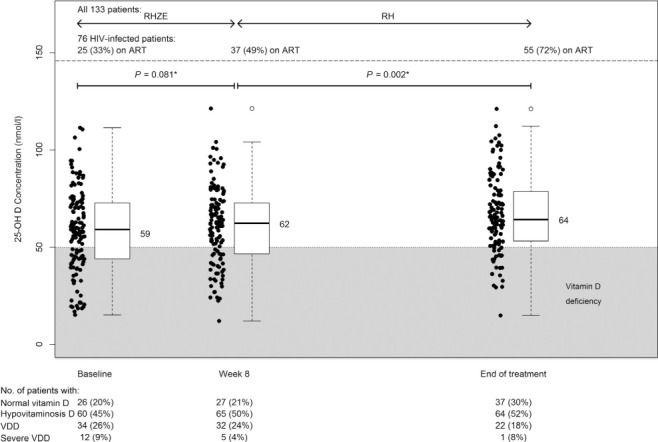
Serum 25(OH)D concentration during anti-tuberculosis treatment. Data only shown for the 133 patients who completed the study. Serum 25(OH)D concentrations gradually increased over time on anti-tuberculosis treatment. Differences between measurements at different times were analysed using paired Wilcoxon tests. R = rifampicin; H = isoniazid; Z = pyrazinamide; E = ethambutol; HIV = human immunodeficiency virus; ART =antiretroviral therapy.

**Figure 6.**
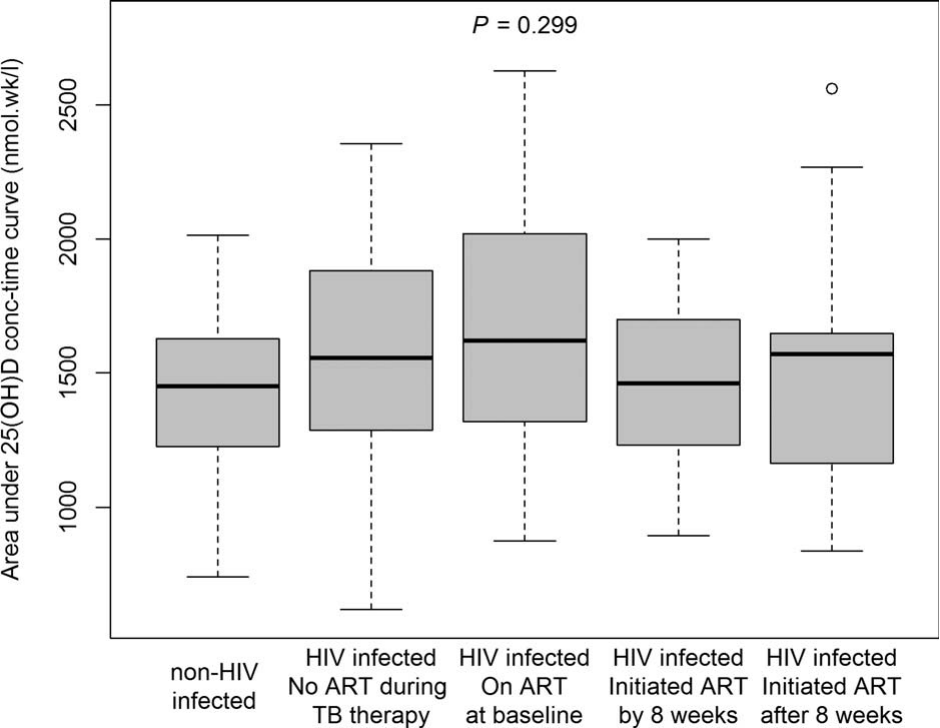
Changes in serum 25(OH)D concentration from baseline to end of treatment in HIV patients by timing of ART initiation. Data only shown for the 133 patients who completed the study. There was no difference in total 25(OH)D exposure, as measured by total time under the concentration-time curve between non-HIV-infected patients and HIV-infected patients, irrespective of the timing of ART initiation during anti-tuberculosis treatment. The *P* value was derived by the Kruskal-Wallis test. HIV = human immunodeficiency virus; ART = antiretroviral therapy; TB = tuberculosis.

Trends in serum 25(OH)D concentration over time were assessed for the 133 patients who reached a final outcome. Median serum 25(OH)D rose to 62 nmol/l by week 8 of treatment and 64 nmol/l by end of treatment (Figure 5). This occurred despite daily administration of RMP and INH to all patients and increased use of ART by HIV-infected participants. Figure 6 shows that 25(OH)D AUC_0–6 months_ was not influenced by HIV status or time of ART initiation. There was insufficient use of ART regimens other than d4T, 3TC and NVP to establish whether different drug combinations influenced 25(OH)D exposure during treatment.

Figure 4 demonstrates that change from baseline 25(OH)D concentration at 8 weeks (OR 1.00, 95%CI 0.97–1.04, *P =* 0.809) or end of treatment (OR 0.991, 95%CI 0.97–1.02, *P*= 0.490) was not associated with final outcome, nor was 25(OH)D AUC_0–6 months_ (OR 1.00, 95%CI 0.99–1.00, *P* = 0.213).

## DISCUSSION

The role of vitamin D in anti-tuberculosis treatment has been subject to prolonged debate. Our data show that in a resource-poor population with high rates of baseline vitamin D deficiency, variability in serum 25(OH)D concentrations did not influence treatment response.

The rate of vitamin D deficiency (39%) among TB patients in our study was slightly lower than that described previously at the same centre in Malawi (42%).^2^ However, the previous study only recruited patients in July, while we recruited all year round, and our vitamin D deficiency rate for July/August was much higher, at 70%. Although the number of daylight hours in Malawi does not change between seasons, a temperature drop from May to August reduces sunlight exposure, as people remain indoors and wear additional layers of clothing. Data from other settings indicate that vitamin D levels drop 1 month after a change in exposure to ultraviolet rays,^23–25^ and our results are consistent with this. Future studies in Malawi should take account of seasonal periodicity.

Other than month of recruitment, most factors linked to baseline 25(OH)D variability were related to dietary or social circumstances (food insecurity, alcohol consumption, low BMI and cooking indoors with biomass fuel). Clinical factors, including HIV status, baseline bacillary load or radiological extent of disease, were not implicated. This broadly corroborates other reports,^3^,^24^,^26^,^27^ and suggests that environmental conditions are the key drivers of vitamin D deficiency.

Overall, vitamin D deficiency rates reported in Malawi are higher than those described in TB patient cohorts in Guinea-Bissau (8.5%),^28^ but lower than those reported in South Africa (80%).^24^ This degree of regional variation underlines the need for caution when extrapolating results of vitamin D studies between African settings.

No analysis of serum 25(OH)D concentrations described a significant relationship between vitamin D status and sputum smear or culture positivity at 2 months or final outcome. This contrasts with previous data from Tanzania, which reported that lower baseline 25(OH)D concentrations were associated with post-treatment relapse.^13^ The reasons for this are unclear, but the overall unfavourable outcome rate was higher in Tanzania (19% vs. 11% in our study), suggesting that there were underlying differences between the cohorts. Furthermore, the Tanzanian cohort had higher baseline 25(OH)D concentrations (median 70 vs. 57 nmol/l), and the main outcome difference was between patients with levels >75 vs. ⩽75 nmol. It is possible that we recruited an insufficient number of patients with 25(OH)D concentrations >75 nmol/l threshold to detect the advantage experienced by this group. However, the results of our study support three clinical trials that showed no benefit of vitamin D supplementation during anti-tuberculosis treatment, despite significant elevation in 25(OH)D concentrations among patients who received supplements.^10–12^

It is reassuring to note that 25(OH)D concentrations increased over time, despite previous reports that RMP, INH and some ART drugs may reduce the bioavailability of active vitamin D metabolites. Improved 25(OH)D concentrations during the course of anti-tuberculosis treatment were also observed in a study in Tanzania.^29^ We propose that increased dietary intake and outdoor activity during recovery from TB contributes to improved vitamin D levels, and adequately compensates any deleterious drug effects on metabolism. It is not possible to definitively confirm this hypothesis with our data, but it does appear that routine 25(OH)D supplementation is not required to prevent exacerbation of baseline deficiency in our setting.

There were several limitations to this work. As only patients with sputum culture-positive TB were included, no comment can be made about vitamin D levels in extra-pulmonary disease. The vitamin D receptor genotype of our patients was unknown, and one clinical trial has suggested that individuals with the *tt* genotype of the TaqI receptor polymorphism are more likely to respond to vitamin D supplementation.^30^ National ART protocols during the study dictated that most patients did not receive EFV, a drug previously associated with vitamin D deficiency.^16^ First-line ART recommendations for TB patients have since changed, and it is possible that the effect of ART on vitamin D metabolism varies by treatment regimen.

## CONCLUSIONS

Vitamin D deficiency remains common among new pulmonary TB patients in Malawi. Social and environmental factors, particularly seasonality, are the main drivers of variability in vitamin D status but this variability does not influence treatment response. Any adverse pharmacological effects of anti-tuberculosis and ART drugs on bioavailability of 25(OH)D are compensated by the positive effects of clinical recovery.

## References

[1] World Health Organization (2014). Global tuberculosis report. WHO/HTM/TB/2014.08.

[2] Banda R, Mhemedi B, Allain T J. (2011). Prevalence of vitamin D deficiency in adult tuberculosis patients at a central hospital in Malawi. Int J Tuberc Lung Dis.

[3] Mastala Y, Nyangulu P, Banda R V, Mhemedi B, White S A, Allain T J. (2013). Vitamin D deficiency in medical patients at a central hospital in Malawi: a comparison with TB patients from a previous study. PLOS ONE.

[4] Rook G A, Steele J, Fraher L (1986). Vitamin D3, gamma interferon, and control of proliferation of Mycobacterium tuberculosis by human monocytes. Immunology.

[5] Rockett K A, Brookes R, Udalova I, Vidal V, Hill A V, Kwiatkowski D. (1998). 1,25-dihydroxyvitamin D3 induces nitric oxide synthase and suppresses growth of Mycobacterium tuberculosis in a human macrophage-like cell line. Infect Immun.

[6] Sly L M, Lopez M, Nauseef W M, Reiner N E. (2001). 1 alpha,25-dihydroxyvitamin D3-induced monocyte antimycobacterial activity is regulated by phosphatidylinositol 3-kinase and mediated by the NADPH-dependent phagocyte oxidase. J Biol Chem.

[7] Liu P T, Stenger S, Li H (2006). Toll-like receptor triggering of a vitamin D-mediated human antimicrobial response. Science.

[8] Martineau A R, Wilkinson K A, Newton S M (2007). IFN-gamma- and TNF-independent vitamin D-inducible human suppression of mycobacteria: the role of cathelicidin LL-37. J Immunol.

[9] Yuk J M, Shin D M, Lee H M (2009). Vitamin D3 induces autophagy in human monocytes/macrophages via cathelicidin. Cell Host Microbe.

[10] Martineau A R, Timms P M, Bothamley G H (2011). High-dose vitamin D3 during intensive-phase antimicrobial treatment of pulmonary tuberculosis: a double-blind randomised controlled trial. Lancet.

[11] Ralph A P, Waramori G, Pontororing G J (2013). L-arginine and vitamin D adjunctive therapies in pulmonary tuberculosis: a randomised, double-blind, placebo-controlled trial. PLOS ONE.

[12] Wejse C, Gomes V F, Rabna P (2009). Vitamin D as supplementary treatment for tuberculosis: a double-blind, randomized, placebo-controlled trial. Am J Respir Crit Care Med.

[13] Mehta S, Mugusi F M, Bosch R J (2013). Vitamin D status and TB treatment outcomes in adult patients in Tanzania: a cohort study. BMJ Open.

[14] Brodie M J, Boobis A R, Hillyard C J, Abeyasekera G, MacIntyre I, Park B K. (1981). Effect of isoniazid on vitamin D metabolism and hepatic monooxygenase activity. Clin Pharmacol Ther.

[15] Brodie M J, Boobis A R, Hillyard C J (1982). Effect of rifampicin and isoniazid on vitamin D metabolism. Clin Pharmacol Ther.

[16] Welz T, Childs K, Ibrahim F (2010). Efavirenz is associated with severe vitamin D deficiency and increased alkaline phosphatase. AIDS.

[17] Lumb R, Van Deun A, Bastian I, Fitz-Gerald M. (2013). Laboratory diagnosis of tuberculosis by sputum microscopy.

[18] Oken M M, Creech R H, Tormey D C (1982). Toxicity and response criteria of the Eastern Cooperative Oncology Group. Am J Clin Oncol.

[19] Ministry of Health Malawi (2008). Guidelines for the use of antiretroviral therapy in Malawi.

[20] Ministry of Health Malawi (2011). Clinical management of HIV in children and adults.

[21] Ministry of Health Malawi (2007). National TB Control Programme Manual.

[22] Holick M F. (2006). Resurrection of vitamin D deficiency and rickets. J Clin Invest.

[23] Pasco J A, Henry M J, Kotowicz M A (2004). Seasonal periodicity of serum vitamin D and parathyroid hormone, bone resorption, and fractures: the Geelong Osteoporosis Study. J Bone Miner Res.

[24] Martineau A R, Nhamoyebonde S, Oni T (2011). Reciprocal seasonal variation in vitamin D status and tuberculosis notifications in Cape Town, South Africa. Proc Natl Acad Sci USA.

[25] Pettifor J M, Moodley G P, Hough F S (1996). The effect of season and latitude on in vitro vitamin D formation by sunlight in South Africa. S Afr Med J.

[26] Friis H, Range N, Pedersen M L (2008). Hypovitaminosis D is common among pulmonary tuberculosis patients in Tanzania but is not explained by the acute phase response. J Nutr.

[27] Nansera D, Graziano F M, Friedman D J, Bobbs M K, Jones A N, Hansen K E. (2011). Vitamin D and calcium levels in Ugandan adults with human immunodeficiency virus and tuberculosis. Int J Tuberc Lung Dis.

[28] Wejse C, Olesen R, Rabna P (2007). Serum 25-hydroxyvitamin D in a West African population of tuberculosis patients and unmatched healthy controls. Am J Clin Nutr.

[29] Tostmann A, Wielders J P, Kibiki G S, Verhoef H, Boeree M J, van der Ven A J. (2010). Serum 25-hydroxy-vitamin D3 concentrations increase during tuberculosis treatment in Tanzania. Int J Tuberc Lung Dis.

[30] Elrefaei M, McElroy M D, Preas C P (2004). Central memory CD4+T cell responses in chronic HIV infection are not restored by antiretroviral therapy. J Immunol.

